# Individual differences in control of language interference in late bilinguals are mainly related to general executive abilities

**DOI:** 10.1186/1744-9081-6-5

**Published:** 2010-01-13

**Authors:** Julia Festman, Antoni Rodriguez-Fornells, Thomas F Münte

**Affiliations:** 1 Department of Neuropsychology, Otto-von-Guericke University, 39106 Magdeburg, Germany; 2 Institució Catalana de Recerca i Estudis Avançats (ICREA) & Dept of Physiology, Faculty of Medicine, University of Barcelona - IDIBELL, 08907, L'Hospitalet (Barcelona), Spain

## Abstract

**Background:**

Recent research based on comparisons between bilinguals and monolinguals postulates that bilingualism enhances cognitive control functions, because the parallel activation of languages necessitates control of interference. In a novel approach we investigated two groups of bilinguals, distinguished by their susceptibility to cross-language interference, asking whether bilinguals with strong language control abilities ("non-switchers") have an advantage in executive functions (inhibition of irrelevant information, problem solving, planning efficiency, generative fluency and self-monitoring) compared to those bilinguals showing weaker language control abilities ("switchers").

**Methods:**

29 late bilinguals (21 women) were evaluated using various cognitive control neuropsychological tests [e.g., Tower of Hanoi, Ruff Figural Fluency Task, Divided Attention, Go/noGo] tapping executive functions as well as four subtests of the Wechsler Adult Intelligence Scale. The analysis involved t-tests (two independent samples). Non-switchers (n = 16) were distinguished from switchers (n = 13) by their performance observed in a bilingual picture-naming task.

**Results:**

The non-switcher group demonstrated a better performance on the Tower of Hanoi and Ruff Figural Fluency task, faster reaction time in a Go/noGo and Divided Attention task, and produced significantly fewer errors in the Tower of Hanoi, Go/noGo, and Divided Attention tasks when compared to the switchers. Non-switchers performed significantly better on two verbal subtests of the Wechsler Adult Intelligence Scale (Information and Similarity), but not on the Performance subtests (Picture Completion, Block Design).

**Conclusions:**

The present results suggest that bilinguals with stronger language control have indeed a cognitive advantage in the administered tests involving executive functions, in particular inhibition, self-monitoring, problem solving, and generative fluency, and in two of the intelligence tests. What remains unclear is the direction of the relationship between executive functions and language control abilities.

## Background

The assumption that a bilingual's two languages have to be processed and dealt with on a constant basis provided that both languages are regularly used (for a recent review, see [1]) implies that the bilingual speaker is forced to control *cross-language interference*, i.e., the unintended use of the current non-target language.

Four lines of research will be reviewed in the following sections, which have contributed to our understanding of the link between bilingualism and *cognitive control *abilities, i.e., the ability to flexibly adapt behavior to current demands by focusing on task-relevant information and behaviors over a period of time while dealing with interference and competition.

### Bilinguals perform better than monolinguals in some respects: Behavioral studies

Since the 1960s, researchers have shown that bilinguals (learners as well as proficient speakers) have cognitive advantages compared to monolingual speakers on a variety of verbal and non-verbal tasks, in particular those involving the resolution of conflicting information and the inhibition of irrelevant information [2-4]; for a review including disadvantages such as smaller vocabulary size, higher number of TOTs, slower picture naming reaction times, see [5]. More recently, it has been demonstrated that bilinguals outperform monolinguals on tasks taxing cognitive control functions, such as the Simon task [6], and the attention network task (ANT) [7]. Explanations have attributed this advantage to greater cognitive control of information processing and attention, and more effective use of cognitive functions [4,8-12]. Superior inhibitory control has been suggested to have developed as a result of bilingual experience [11,13,14]. Additionally, recent research implies earlier development of executive function in bilingual children (around the age of 3 years) as compared to monolinguals (at approximately 4-5 years of age) [10,15-17].

We suggest that individual differences should be considered more thoroughly (see below). In addition to different levels of proficiency, several factors (e.g., the studied age range, language typology, task differences, sociodemographic variables, location of research in conjunction with language status, etc.) may explain replication difficulties of effects of bilingual cognitive advantages (e.g., [18]; for a recent review, see [5]). To summarize, bilinguals' cognitive advantages have been demonstrated, but the underlying mechanisms are not yet sufficiently understood, and factors influencing performance differences need to be investigated more systematically.

### Areas involved in language switching and language control: Imaging studies

Neuroimaging studies have shown that a wide neuronal network is involved in control over *language selection *("*language choice*") and *switching *(i.e., use of language A, then of language B) (for a review, see [19,20]). For example, in an early PET-study by Price, Green and von Studnitz [21] the left inferior frontal region and bilateral supramarginal gyri were more highly activated during switching between words from German and English in a translation task in proficient bilinguals. In two block-design fMRI-studies [22-24] on language switching, Spanish-English bilinguals were required to switch between both languages in a picture naming task. Compared to single language blocks, a higher activation was reported in left dorsolateral prefrontal cortex (DLPFC), i.e., one of the core executive control areas, and premotor areas in mixed language blocks. Hernandez [24] suggested recently that the observed increased activation during the mixed condition in the precentral gyrus, which has been associated to phonological processing, may reflect phonological interference across languages in early bilinguals.

In an fMRI-study by Rodriguez-Fornells and colleagues [25] interference was observed in bilinguals in a language production task reflected in particular by activation of left prefrontal cortex and the supplementary motor cortex in noGo-trials. These areas have been associated to executive and cognitive control functioning, which, according to Smith and Jonides [26], reflect a heterogeneous group of higher order "meta-cognitive" functions that are needed to orchestrate and supervise the behavior of humans, such as the selection of response alternatives [27,28], inhibition of irrelevant information stored in working memory [29] and control of task switching [30,31].

In a recent fMRI study Crinion and colleagues [32] specifically studied the regions involved in language control in German-English and Japanese-English subjects. Using a semantic priming task, the results showed a language-dependent activation at the head of the left caudate nucleus: semantically related and from the same language prime-target word pairs reduced activation in the left caudate nucleus, but not when these pairs were from different languages. Crinion et al. suggested that the caudate nucleus might play a crucial role in *language choice*, i.e., determining which language should be used for production and *language control*, i.e., controlling language output by checking to which language the selected words belong (cf. [33]). Supporting evidence comes from a recent study by Abutalebi and colleagues [34] using a picture naming task in different mono- and bilingual conditions. Increased activation was found in the left caudate and the anterior cingulate cortex (ACC) in conditions when both languages had to remain active.

The results observed in these studies demonstrate that language control, language choice and switching rely on interrelated processes predominantly engaging frontal-subcortical regions. Some of these regions have been associated with executive functions and cognitive control in a variety of tasks, what in turn suggests that language control might be implemented by recruiting such domain general higher control functions.

### Pathological language switching: Case reports

A case report on an Italian engineer (L1 Friulian, L2 Italian) provides a special case demonstrating the loss of control over language choice [35]. The patient with a left prefrontal and medial frontal tumor was no longer able to control socially appropriate switching between languages. He switched across different utterances (pathological switching without aphasia), in contrast to pathological mixing (aphasia), which is identified by switches within the same utterance. Neuropsychological tests indicated that his linguistic behavior was independent from intellectual, attentional or praxic disorders. Two other case studies report on pathological switching and mixing (involving aphasia) after a subcortical infarction [36] and a posterior left thalamic hemorrhage with recurrent bleeding [37]. These findings point again at the role recently attributed to subcortical structures in language selection and language switching [32,33].

### Interference effects: Psycholinguistic and electrophysiological studies

According to Green [38] errors of interference are considered to be failures of control over target language production. Cross-language interference is usually defined as the **unintended **use of the current non-target language during target-language production (e.g., the speaker intended to say the word "Baum" in German, his L1, but produced it instead in his L2, English, and said "tree". This involuntary production of the word in L2 rather than in L1, against the speaker's language choice, has to be differentiated from another linguistic phenomenon, namely *code switching*, i.e., an **intentional **switch to the other language according to the speaker's language choice). Interference during production is avoided in "normal" language processing due to sufficient and unimpaired control mechanisms. Factors such as mental fatigue and stress have a negative impact on the speaker's control ability [39] so that cross-language interference is increased.

Bilingual picture-word interference paradigms provide evidence for the activation of both languages, since target naming is faster when the distractor word is the translation equivalent of the target (e.g., [40-42]). Recent picture-word interference studies claimed that the parallel activation is not limited to stages of lexical selection, but lasts until the stage of phonological representation [43].

Additional evidence for phonological interference is provided by a combined event-related potential (ERP) and fMRI study [25]. In a tacit Go/noGo-picture naming paradigm, balanced German-Spanish bilinguals had to decide whether the target word of the presented object started with a consonant or a vowel. Behavioral results showed that phonological interference impaired processing of the target language: bilingual participants made more errors and reacted slower on non-coincidence trials (when the target word started in one language with a consonant, but in the other with a vowel) than in coincidence trials. When comparing the overall response latencies between the bilingual and the monolingual control group in the ERP-experiment, bilinguals were about 200 ms longer. This could be interpreted as reflecting the need to engage control processes in order to be able to name pictures in a monolingual setting. Interference also manifested itself in the ERP-analysis in the form of an increased frontal negativity for non-coincidence trials in Spanish and German naming blocks (see also [25]). A very similar pattern in terms of performance and ERPs was observed in a follow-up study addressing gender interference across languages [20].

In sum, evidence from different methods reviewed here indicates that during bilingual processing both languages are activated and cause interference. Inadvertent production of non-target-language words, however, is, in most cases, prevented by the speaker's control system. Parallel activation appears to be an additional processing load for bilinguals, implying an overall negative impact on processing speed and mental resources compared to monolingual processing. On the other hand, bilinguals were found to have a cognitive advantage over monolinguals due to excessive practice of language control in tests involving conflict resolution and distracter inhibition. Imaging studies revealed that the same areas are involved in language control and executive functions, indicating that bilingual language use might engage executive control (already at lower levels of L2-proficiency, see [44]).

### Individual differences in inadvertent language switching

In bilingual research, bilingual speakers are characterized by criteria such as age of acquisition (AOA) of the second language, instruction (formal or informal), frequency of use of both languages. Furthermore, levels of proficiency in different skills in both languages (speaking, reading, writing, and listening) are assessed for participant description and matched for group comparisons. In the present study, we suggest the existence of an additional factor that might influence bilingual language production but has heretofore been largely ignored: individual differences in *language control abilities*. The concept of language choice assumes that the speaker is able to choose at any point in time which and how many languages he wants to use [38]. We suggest that inadvertent language switching as well as failure to switch languages when necessary reflect the speaker's language control abilities.

Based on the anatomical-functional evidence that there is a link between language control and executive control, and the necessity for such a relation on behavioral and electrophysiological grounds, we hypothesize that individual language control abilities and susceptibility to interference in bilinguals might be related to individual differences in executive brain functions. To this end we assessed individual differences in rates of cross-language interference, reflecting language control problems, and thus included both switches to the "wrong" target language as well as failures to switch to the "correct" target language. We divided the sample studied in two groups, "switchers" and "non-switchers". This group division was made based on their performance (i.e., the number of cross-language interference) in a bilingual picture naming task in which we employed the alternating-runs paradigm [45]. We suggest that individual differences in language control abilities as determined here might be reflected in everyday life: in a conversation in which two languages are used, the switcher might switch spontaneously or following previous switches of his communicative partner, whereas a non-switcher might stick to his initially chosen language of communication for the entire length of his/her conversation.

In order to investigate whether individual differences in language control abilities are related to more general cognitive control abilities (e.g., inhibition), a number of neuropsychological tasks devised to measure executive functions as well as standard measures of intelligence were administered.

## Methods

This study had been approved by the ethics committee of the Otto-von-Guericke University, Magdeburg, and was performed in accordance with the ethical standards laid down in the 1964 Declaration of Helsinki.

### Participants

Participants were recruited by an advertisement at the University of Magdeburg, gave their informed consent prior to inclusion in the study and were paid for their participation.

For the purpose of screening participants with regard to their language proficiency in both languages (Russian, German) and their language background we used a bilingual picture naming task and a language background questionnaire (see below) in a 1^st ^session lasting about 60 minutes. The mean of all produced errors in the picture naming task was 51.2 (SD = 17.6; 240 trials per subject). The number of errors produced by 19 of our initial 49 participants was higher than the estimated upper confidence interval (56.2). Apparently, the proficiency level in at least one of the languages was quite low for these subjects; they were excluded from analysis and further participation. Another subject dropped out and reduced the final sample size to 29 participants (21 women). Their ages ranged from 17 to 45 years (M = 24.7, SD = 5.09) (note that only one subjects was 45 years old, the others were maximally 30 years old).

According to the questionnaire (section on language history, adapted from [46]), 28 participants spoke Russian as their first language (L1) and German as L2 (for one participant German was L1 and Russian L2). Most participants were late bilinguals, exposed to German at an average of 11.4 years (SD = 6.1). They had been living in Germany for an average of 9 years (SD = 4.1), and were on average 15.5 years old (SD = 6.5) upon arrival to Germany. All participants were living in Magdeburg, Germany, at the time of testing, and were regularly exposed to German and Russian. Most participants were students at the university (n = 16), some were still in high school (n = 9), and some had already finished their university studies (n = 4).

In the self-rating section in the same questionnaire, participants rated their current proficiency in four language skills (speaking, comprehension, writing and reading) for all of their acquired languages on a 4-point scale (1 = poor, 2 = moderate, 3 = good, 4 = perfect). On average, participants rated themselves equally proficient in speaking Russian (M = 3.5; SD = 0.63) and speaking German (M = 3.3; SD = 0.66); their rating indicated good (with a trend to perfect) language proficiency (see Table 1).

**Table 1:** Self-ratings of language skills in both languages

	*Russian*		*German*	
**Skills**	***Switcher***	***Non-Switcher***	***Switcher***	***Non-Switcher***
	
speaking	3.5 (.5)	3.4 (.7)	3.2 (.8)	3.5 (.5)
reading	3.9 (.4)	3.5 (.7)	3.4 (.7)	3.8 (.4)*
writing	3.3 (.8)	2.9 (1.1)	2.7 (.9)	3.6 (.5) **
comprehension	3.9 (.4)	3.7 (.5)	3.5 (.5)	3.8 (.4)

Participants of both groups provided self-ratings for 4 language skills in both languages on a 4-point scale (1 = poor, 2 = moderate, 3 = good, 4 = perfect). Means and standard deviation (SD) are reported. Significant differences between groups are indicated by an asterisk with * = p < .05 and ** = p < .01.

### Methods and Procedures

#### 1st session: Bilingual Picture Naming Task [45] and Language Background Questionnaire (adapted from [46])

The bilingual picture naming task was used to create two groups of bilinguals, "switchers" and "non-switchers". In this task, bilinguals are faced with a particular difficulty compared to monolinguals: due to parallel activation of words from both languages, bilinguals have to suppress non-target language words that may pop up during the stages of lexical search and retrieval. Moreover, the task demands vary depending on the proficiency level in both languages. In sum, in a bilingual setting, this task measures language proficiency in the sense of lexical competence, and additionally, it provides an indication of the speaker's ability to prevent interference.

For this task, 240 pictures (+48 for practice) of common objects were selected from the Snodgrass and Vanderwart [47] set comprising black line drawings on white background. Word frequency (i.e., lemma frequency per million) was determined for German with CELEX [48], and for Russian with an on-line frequency dictionary [49]). Pictures with a one-to-one correspondence in Russian and German were included (e.g., German "Apfel", Russian "яблоко" for English apple). The following types of pictures were excluded: if they were cognates in both languages, culturally specific (e.g., eskimo, banjo), or had no one-word translation to Russian (e.g., ambulance, typewriter). Pictures that had alternative names in German or Russian (e.g., Möhre and Karotte for English carrot) were only used as practice items.

Each participant was tested individually in one 30 minute experimental session (1^st ^part of screening session). Subjects were seated in a dimly lit, sound-attenuated room in front of a computer screen. All stimuli were displayed in the middle of a high-resolution screen. Viewing distance was approximately 60 cm. The DMDX program [50] controlled the display of the visual stimuli and measured speech-onset latencies. All verbal responses were recorded. Participants were asked to name the pictures as fast as possible (trying neither to make errors nor to correct themselves). They were informed that the language in which a given picture had to be named was determined by the color (red or green) of a frame, which appeared prior to the picture, and that two pictures in a row required a response in German, and the next two in Russian, and so on (GG RR GG RR).

Each trial had the following structure: First, a fixation cross appeared in the center of the screen for 100 ms. Then, a colored frame (red or green) was displayed that surrounded the fixation point. After 300 ms the stimulus picture was shown in the frame for 1500 ms. The next trial started after 1500 ms with the fixation point. The experiment consisted of six blocks, each with 40 pictures (20 per language). Before each experimental block, 8 practice trials were administered. Between the blocks, the participant was allowed to rest. The presentation order of the pictures was fully randomized. The association of one color with one response language was counterbalanced: half of the participants responded to a green frame with German, and half with Russian. Half of the subjects started with German, the others with Russian.

After completing the picture naming task, subjects were asked to fill in the questionnaire (adapted from [46]) in German, providing information on language background, acquisition history, language use and self-ratings of language proficiency. This second part of the 1^st ^session lasted about 30 minutes and took place in a silent laboratory room.

#### 2^nd ^Session: Executive functions tasks and intelligence tests

Four executive function tasks (Tower of Hanoi, Go/noGo, Divided Attention, and Ruff Figural Fluency Test) as well as four subtests of the German version of the Wechsler Adult Intelligence Scale (WAIS) were administered in one session (lasting about 90 minutes), about 3 weeks after the screening session. For the computerized tests, each participant was tested individually in a dimly lit, sound-attenuated room in front of a computer screen. All stimuli were displayed in the middle of a high-resolution screen. Paper-and-pencil tests were administered in a silent laboratory room. The sequence of tasks was the same for all subjects, alternating neuropsychological and intelligence tests to avoid fatigue.

##### Tower of Hanoi (TOH)

The participant's goal in the TOH puzzle was to move all the discs from the left to the right peg. The subject was seated in front of a computer screen and clicked and dragged one disc at the time with the mouse button in a computerized version http://www.osborn-software.de. A disc could only be placed either on an empty peg, such as the middle when starting, or on top of a larger disc. Every rule violation was punished with 100 error points. Subjects were asked to complete the task as quickly as possible by using the fewest possible moves. For training purposes, three discs were used; subjects had to rearrange four and five discs for testing.

Solving this test involves several aspects of executive functions: *problem solving*, *working memory*, and *inhibition *[51-53]. Whether this task provides a true measure of the ability to plan has become a matter of debate (see [54] for an argument for procedural repetitive trial-and-error learning rather than "look ahead" planning). A recent structural equation modelling study showed that inhibition contributed more than working memory (information updating and monitoring) to explain the performance observed in the TOH task [55], for example overcoming the tendency not to use "conflict moves" such as blocking the goal peg with a disk that must later be cleared [54].

For the analyses, two measures were used. As an indicator for the efficiency to solve a problem, we used the number of moves. The error points were meant to indicate inhibition abilities in accordance with specific rules. Data for five discs were incomplete, because some of the subjects could not complete the task.

##### Go/noGo Paradigm

The ability to deliberately *inhibit *dominant, automatic, or prepotent responses when necessary was measured in a Go/noGo task. This executive function component has been associated to the right medial and inferior frontal cortex [56-58]. A typical visual Go/noGo paradigm (based on the "Test battery for Attentional Performance" [59,60]) was employed. Five different white stimulus patterns (e.g., parallel rows of dashed lines) were presented successively in random order in the center of a black screen for 1000 ms each. A total of 100 stimuli were displayed, of which 40 were "Go"-stimuli. A Go response (button press) was required only on two of the five stimuli patterns; a noGo response (withhold button press) on the three other patterns. *Working memory *was involved as well, because the stimuli had to be remembered. For the analyses, response latencies as well as percentage of false alarms (as a measure of failed inhibition) were calculated.

##### Divided Attention

Based on the test "Geteilte Aufmerksamkeit" from the "Test battery for Attentional Performance" [59,60] a visual and an auditory task were created. In the visual task, the participant was presented with a 4 × 4 matrix consisting of white crosses and dots on black background on a computer screen. The participant had to identify whether four crosses form a square at any point within the matrix (target stimulus). In the test, 100 white stimuli pictures were displayed on a black screen, each for 2000 ms, and then replaced by the next stimulus picture. In the auditory task, two different sine-wave tones (high = 865 Hz, and low = 800 Hz) were presented to the participant wearing head phones. While listening to the sequence of tones, the participant was asked to identify occasional tone repetitions (target stimulus); otherwise, the tones alternated regularly, every 1000 ms. Two hundred auditory stimuli were presented.

In a short training phase, the loudness was adjusted individually. Participants were required to respond to visual and auditory targets as quickly and correctly as possible by button press. For the analyses, response latencies as well as percentage of hits and false alarms were calculated. This dual task situation necessitates the adequate allocation of attentional resources to each of the two tasks and thus probes executive aspects of attention.

##### Ruff Figural Fluency Test (RFFT)

This paper-and-pencil test assesses *generative fluency*, another aspect of executive functioning [61], requires the generation of **novel **designs and appears to be associated predominantly with right dorsolateral prefrontal cortex (for a review on the double dissociation between verbal/figural fluency and left/right hemispheric function, see [61]). Successful task performance relies on *fluent and flexible thinking*, i.e., the formulation and use of production strategies (e.g. enumeration, rotation) involving *self-monitoring *to avoid repetition of responses [62], i.e., *inhibit *repeating previously generated responses [63], while observing the generation rule. More specifically, the RFFT [64,65] consists of five subtests, each of which contains a different stimulus pattern made up of five points. Every subtest (1 minute) is administered on a separate sheet of paper, each with 35 identical stimulus patterns presented in a 5 × 7 matrix. In the second and third subtest, the arrangement of the points of the first one (forming a circle) is presented with distracters (i.e., interference patterns). Part 4 and 5 represent variations of Part 1, where the points are asymmetrically positioned, but all squares are alike on each page. The number of produced unique patterns as well as the error ratio (perseverations divided by the sum of unique patterns) was scored.

##### Subtests of the WAIS-R

We used the following subtests from the WAIS-R (German adaptation HAWIE-R, [66]): "Information", "Similarities", "Picture Completion", and "Block Design". The first two subtests are included in the Verbal IQ test scale, whereas the latter two are used to analyze non-verbal capabilities (Performance IQ test scale). The first one, Information, examines the participant's general knowledge and is focused on the capability to understand simple information, i.e., short test questions, correctly. The second verbal test, Similarities, assesses logical reasoning, in particular abstraction and conceptualization. The participant is presented verbally with item pairs (e.g., banana and orange; library and zoo), and the abstract similarity amongst them has to be identified. The third subtest, Picture Completion, assesses aspects of visual perception, i.e., the ability to differentiate between important and unimportant details. The task consists of identifying a missing feature in a number of line drawings [67]. Finally, the Block Design, is supposed to reflect the participant's visual-constructive and problem-solving abilities. The participant is required to organize blocks according to patterns on cards.

### Data Analysis

Since we did not collect data from patients, but rather healthy bilingual participants, we used the raw scores for statistical analyses, and not normalized results. The results of all the tests described below were subjected to a test for normality (Shapiro-Wilk's test). Normally distributed results were further analyzed with *t*-tests (two independent samples). No MANOVA was performed, because we tested the same null-hypothesis in all experiments.

## Results

### Establishing two groups, language switchers and non-switchers

For the creation of both groups, errors of interference were scored when the correct name was uttered in the **non-target **language in the picture naming task. Using Ward's method, participants were grouped into 2 clusters on the basis of these errors, "switchers" (n = 13, 11 women, 10 to 20 errors or interference) and "non-switchers" (n = 16, 10 women, 0 to 5). Both groups are further described and characterized in Tables 1, 2, and 3. Regarding language use patterns, group comparisons yielded significant differences only in the use of two domains: non-switchers used more German with their brothers and sisters than switchers (more Russian), and Russian and German almost equally with other relatives, whereas switchers used mostly Russian. Since these two domains should constitute rather small fractions of all-day long communication and language use within the larger family, the observed differences do not seem to have a sufficient impact on language behavior in order to explain interference differences in the picture naming task.

**Table 2:** Possibly confounding factors

**Factors**	***Switcher***	***Non-Switcher***
	
age	26.4 (6.7)	23.4 (3.1)
number of languages used	3.2 (.8)	3.5 (.6)
number of years spent in Germany	7.9 (3.2)	10.3 (4.5)
age at acquisition of L2 German	12.9 (8.1)	10.5 (4.1)

Means and standard deviation (SD) are reported for both groups. Group differences were not significant.

**Table 3:** Language performance of both groups in two language tests

**Tasks and performance measures**	***Switcher***	***Non-Switcher***
	
**Verbal Fluency**		
**German**		
category FOOD correct	20.6 (4.5)	21.8 (4.6)
category CLOTHES correct	18.1 (4.9)	18.8 (4.9)
letter S correct	10.2 (4.4)	12.1 (4.5)
letter H correct	8.8 (2.5)	9.5 (3.4)
**Russian**		
category ANIMALS correct	17.7 (5.0)	20.6 (6.1)
category PLANTS & FLOWERS correct	17.1 (7.1)	16.1 (6.9)
letter P correct	11.8 (5.5)	13.8 (4.6)
letter R correct	11.9 (3.3)	11.7 (4.9)
Errors of interference total	1.1 (1.3)	.3 (.7) *
**Interview**		
Errors of interference in German	.6 (1.0)	0 (0.0) **
Errors of interference in Russian	10.8 (10.5)	3.3 (4.8) **

Means and standard deviation (SD) are reported for performance on two bilingual language tests of both groups. Significant differences between groups are indicated by an asterisk with * = p < .05 and ** = p < .01.

We found no significant group difference in other possibly relevant factors such as age, age at acquisition of L2 German (after L1 Russian), and number of years spent in Germany, etc. (see Table 2). In both groups were more women than men. Both groups did also not differ on the educational component of socio-economic status, since all subjects were in their last year of high-school or already had completed at least high-school or more. With regard to their professional life, subjects were mainly students, or a teacher, translator, economic engineer, accountant, or businesswoman, indicating their professional integration in Germany.

Language proficiency difference, however, could be suggested to be the most likely candidate to explain interference differences in the picture naming task. In general, error rates, in particular broken down into error categories (i.e., no response, within-language substitution, interference) show, that the only difference between the languages was the frequency of within-language substitution, i.e., when a word semantically or phonologically similar to the target was produced (German 9%, Russian 6%; no response 11% in each language, interference 4% in each language). Based on these data, we suggest that the interference score was not primarily a function of switching into German. To exclude the possibility that interference was due to lack of knowledge, a post-hoc assessment of errors of interference revealed that 95% of those errors in German and 98% in Russian were known correctly. We suggest that unintentional switches were not psycholinguistic code-switches.

As regards the self-rating of language skills (Table 1), the best indication for self-assessed task-relevant proficiency was attributed to "Speaking". Although the difference in proficiency for reading in German and for writing in German were significant between both groups, it was less relevant here, because the task of picture naming as well as the WAIS was a spoken task and did not entail reading or writing skills.

Further objective measures of language proficiency were used (in later sessions with the same subjects), including a bilingual verbal fluency test, and a bilingual interview; they are reported in detail elsewhere, a paper focused on language proficiency measurements of both groups (Festman, Rodriguez-Fornells, & Münte: Language control abilities of late bilinguals, submitted). The bilingual verbal fluency task [45] involved retrieval of responses in one of both languages to category and letter stimuli (per language two category and two letter stimuli were presented). Both groups performed the same as to the number of correct responses in both languages (Table 3), but switchers produced significantly more errors of interference than non-switchers. The switcher vs. non-switcher group distinction based on the naming paradigm results also held up under more natural circumstances. In a bilingual interview (target language alternation every 5 min, lasting 30 min) with **the same subjects **the switchers produced significantly more errors of cross-language interference in both languages than the non-switchers. Group differences were highly significant for both languages (see Table 3). Speech production, however, did not differ in any other aspect, e.g., fluency or word-finding difficulties. In sum, considering all the previous results, both bilingual groups mostly differed in their inability to prevent errors of cross-language interference in monolingual settings, i.e., the use of the non-target language when target language use is required by the experimental setting.

### Executive Functions Tasks

The results of the four neuropsychological tests administered for both groups, switchers and non-switchers, are presented in Table 4. When completing the **Tower of Hanoi (TOH) **task, switchers needed significantly more moves and received more error points. This result suggests a more efficient behavior in non-switchers when compared to the switcher group. In the inhibition **Go/noGo **task, switchers were significantly slower, but in spite of this, they also made more errors of commission on noGo-trials. These results suggest better inhibitory control in the non-switchers. Similarly, in the **Divided Attention **task, switchers were also slower and less accurate than non-switchers. Finally, in the generative fluency non-verbal **RFFT **task, the switcher group produced significantly fewer unique designs than the non-switch group, indicating a less efficient strategy. The error ratio, an indicator of planning efficiency which is calculated dividing the number of perseveration errors by the sum of unique designs, was significantly higher for the switcher compared to the non-switcher group. The non-switcher group was better able to minimize repetition errors and to maximize the number of unique patterns.

**Table 4:** Results of neuropsychological tests for both groups

**Tasks and performance measures**	***Switcher***	***Non-Switcher***
	
**TOH**		
moves	43.8 (11.7)	29.3 (12.8) **
error points	515 (684)	119 (172) *
**Go/noGo**		
RT Go	551 ms (81 ms)	503 ms (58 ms) *
false alarms	9.1% (8.6)	3.3% (3.0) **
**Divided Attention**		
RT correct	742 ms (89 ms)	690 ms (67 ms) *
responses correct	79.1% (4.5)	86.7% (3.0) **
**RFFT**		
unique designs	80.2 (23.1)	94.6 (22.7) *
mean of error ratio	.25 (.2)	.076 (.08) **

Means and standard deviation (SD) are reported for performance on four neuropsychological tests of both groups. Significant differences between groups are indicated by an asterisk with * = p < .05 and ** = p < .01.

### Intelligence Subtest

In relation to the two verbal subtests, the language switcher group produced significantly fewer correct responses in the **Information **subtests than the non-switch group. This implies that the non-switch group was better at providing the most appropriate and accurate information with regard to the question in the given amount of time (see Table 5). Similarly, in the **Similarities **subtest, the switcher group produced significantly fewer correct responses than the non-switchers. This result shows that the non-switcher group had a cognitive advantage over the switcher group with regard to both verbal sub-tests.

**Table 5:** Results of intelligence tests for both groups

**WAIS-Subtests**	***Switcher***	***Non-Switcher***
	
Information	13.4 (3.3)	17.2 (3.3) **
Similarities	22.1 (4.1)	26.1 (3.9) **
Picture Completion	14.4 (1.6)	15.1 (1.3)
Block Design	35.4 (9.6)	38.13 (7.9)

Means and standard deviation (SD) are reported for performance (correct responses) on intelligence tests of both groups. Significant differences between groups are indicated by an asterisk with ** = p < .01.

In contrast to that, in the two subtests of the Performance IQ, both groups produced a similar number of correct responses in the **Picture Completion **task. Regarding the **Block Design **subtest, the switcher group received fewer points than the non-switch group, but this difference was not significant.

## Discussion

### Resistance to language interference and its link to executive functions

We asked whether bilinguals' individual susceptibility to unintended intrusions could be explained by individual differences in executive functions. We found that individual differences in susceptibility to interference, measured according to language inhibition abilities in bilingual tasks (picture naming, verbal fluency, interview), were indeed reflected in task performance differences of switchers and non-switchers in all of the four tasks tapping executive functions as well as in two subtests of the intelligence test, despite the small number of subjects. More specifically, results show that those subjects (non-switcher group) with better interference control in the language tasks performed also better in the neuropsychological tasks: The non-switcher group demonstrated a significantly better performance on the TOH (fewer moves to reach the target position) and RFFT (more unique patterns) task. They responded significantly faster (Go/noGo and Divided Attention - response latencies). Non-switchers produced significantly fewer errors in the TOH (error points), Go/noGo (false alarms), and Divided Attention (false alarms and misses) than the switcher group. Results of the RFFT (error ratio) further indicated that they were better able to minimize repetition errors while maximizing unique responses.

As the four tasks chosen here tap specific executive functions, a difference in executive brain functions likely explains the observed group differences in the performance on these tasks. In the following, we will discuss whether performance differences can be related to specific executive functions, i.e., *inhibition *and *performance monitoring*, *problem solving*, *generative fluency *and *planning efficiency*. (*Working memory *is the executive function which is inherent to each of the tasks, but has not been measured in isolation here and will therefore not be considered further in the discussion.) Firstly, results indicate non-switchers might have better abilities to inhibit (i) rule-breaking actions (TOH), (ii) distracting, conflicting stimuli (Go/noGo, Divided Attention) and thus reducing response conflict (between stimuli in Go/noGo and between tasks in Divided Attention), facilitating response selection and response execution on Go-trials (faster RTs in Go/noGo, and Divided Attention), (iii) motor responses to information irrelevant for the current task such as noGo-stimuli (false alarms in Go/noGo). The non-switchers' better ability to monitor their performance (avoidance of repetition errors in RFFT, and of misplacing disks in TOH, monitoring of motor responses on Go-trials in Go/noGo and Divided Attention, and focus on the relevant task set in Divided Attention) is closely related to inhibition, because that seems to be the underlying mechanisms to execute monitoring. Secondly, results suggest improved problem-solving (TOH) and generative-fluency skills (RFFT) as well as better planning efficiency (RFFT) in non-switchers.

Interestingly, the performance of the non-switcher group was **not **characterized by a speed-accuracy-trade off (i.e., despite faster response latencies, the accuracy of performance did **not **decrease in this group); they outperformed the switcher group both in speed and accuracy of their behavior. It could be argued that enhanced attention to task requirements and control over behavior are possible in this bilingual group due to more structured, efficient and controlled information processing.

### Interplay of executive functions and language control abilities

Our data clearly imply a close relation between switch behavior and executive functions in bilinguals. Bialystok and colleagues [6] suggested that bilingualism boosts inhibitory control mechanisms, and that bilingualism results in greater use of inhibitory control, because it is invoked every time language is used [4]. Kovács [68] is even more specific and limits the training effect of bilingualism to the response level of the inhibitory system, meaning that there is an advantage due to training in selecting and inhibiting competing responses for language output/motor response. In a similar vein, Kroll and colleagues [1] suggested that bilinguals have not learned to avoid cross-language competition but trained to deal with the competition.

Our data tackle the relationship between executive functions and bilingualism from a different angle. Those bilinguals with better executive abilities demonstrated better language control abilities: they were less prone to switch involuntarily or to fail switching. The question arises whether differences in executive functions in bilinguals as demonstrated in our switcher and non-switcher groups could be the result of differences in L2-acquisition or language practice. This implies that language separation during L2-acquisition might be emphasized for non-switchers, whereas for switchers less emphasis is put on constant monitoring of their speech output. These differences might be due to parental and peer group influences and may also depend on the language environment.

Alternatively, it might be that pre-existing differences in executive functions shape the language control abilities of late bilinguals. Recent studies have shown that the executive control of cognition and action has a high heritability [69]. Disentangling training from genetic influences on executive attention, Rosario Rueda and colleagues [70] claimed that executive attention develops under strong genetic impact. One could thus speculate that individual differences in inadvertent language switching are a consequence of genetic differences in executive functions rather than a result of environmental influences. Obviously, an answer to the question of the direction of the relationship between executive functions and language control abilities would have great implications for the practice of learning a new language and therefore should be studied using longitudinal designs.

### Language, cognitive control and intelligence

Interestingly, switchers and non-switchers also differed on two out of four subtests of the Wechsler-Intelligence Scale (German Version) which were administered to examine whether there is a link between resistance to interference in the language task and aspects of intelligence. Miyake and colleagues [55] showed that inhibiting of prepotent responses, updating working memory representations and shifting between tasks or mental sets are three separable executive functions. Following this line of research, Friedman et al. [71] reported that in their study, only updating working memory was related to both "fluid intelligence" (Gf, reflecting higher mental abilities) and "crystallized intelligence" (Gc, indicating acquired knowledge) but not inhibition or shifting.

We suggest that good executive control, in particular suppression of irrelevant and conflicting information, facilitated and thus speeded up response retrieval in the WAIS. Non-switchers could faster retrieve the most appropriate response (Information subtest) and establish the most fitting mental connection between the two verbal stimulus items (Similarity subtest). In contrast, both pictorial, nonverbal tasks did not rely on inhibition to such extent and thus did not reveal group differences.

### Individual differences in inhibition

In our study, two groups of bilingual subjects were grouped according to their different language control abilities in a bilingual naming task. Other tasks assessing interference, such as the Stroop test, have been used earlier [72] to classify subjects as being either relatively resistant or more susceptible to interference. The more-resistant group did perform better on other tasks that involved interference. Sommer and colleagues [73] claimed that the efficiency of inhibiting prepotent responses as measured by cognitive tests differs between individuals, but moreover also demonstrated that inhibitory efficiency as measured by the Stroop-test and the Attention Network Test (ANT) did not correlate. Barkley [74] defined three forms of inhibition: inhibition of a prepotent response, of an ongoing response, and interference control. Nigg [75] proposed to limit the taxonomy to inhibition of a prepotent response and interference control, with the first to be measured with a Stop Paradigm, and the second with Stroop or Flanker tasks. Nigg's distinction is based on different neural circuits involved in inhibition of prepotent responses (lateral and orbital prefrontal cortex), and interference control (anterior cingulate, DLPFC and basal ganglia). Friedman and Miyake [76] stressed that prepotent response inhibition and resistance to distractor interference were similarly involved in tasks such as random number generation, task-switching, and everyday failures; however, resistance to proactive interference (old material interferes with recollection of current stimuli) was found to be related to reading span recall and unwanted intrusive thoughts. Recently, Martin-Rhee & Bialystok [77] applied the distinction between types of inhibitory control ("interference control" vs. "response inhibition") comparing mono- and bilingual children and demonstrated a bilingual advantage for interference control, but not for response inhibition.

With regard to the present study in which two bilingual groups were compared, it appears that language control abilities were related to executive functions in general rather than to specific aspects of inhibition. More research is necessary to determine even more precisely the behavioral and functional underpinnings of the observed individual differences between our two groups. An intriguing question remains whether there is a difference in the degree of executive control, that is employed [20] between our two groups, or the efficiency with which it is applied [6].

## Conclusions

Language control abilities of late Russian/German bilinguals were reliably associated with executive functions. Establishing the directionality of this relationship in further studies should be made a research priority.

## Competing interests

The authors declare that they have no competing interests.

## Authors' contributions

JF conducted data collection, data analysis, literature review and prepared all drafts of the manuscript; TFM significantly contributed to draft revision; ARF contributed to theoretical interpretation. All authors have read and approved the final manuscript.
